# Effects of Smartphone-Based Hospital-Family Transitional Care on Symptom Burden and Quality of Life in Elderly Patients with Depression

**DOI:** 10.31083/AP39894

**Published:** 2025-04-22

**Authors:** Jianghong Tang, Shilin Zhu, Yuxiang Huang

**Affiliations:** ^1^Department of Geriatrics, The Second Affiliated Hospital of Hunan University of Chinese Medicine, 410005 Changsha, Hunan, China; ^2^Department of Nursing, The Second Affiliated Hospital of Hunan University of Chinese Medicine, 410005 Changsha, Hunan, China

**Keywords:** transitional care, quality of life, geriatric depression, symptom burden

## Abstract

**Objective:**

To explore the effects of smartphone-based hospital-family transitional care on symptom burden and quality of life in elderly patients with depression.

**Methods:**

This study retrospective analyzed the clinical data of 168 elderly patients with depression admitted to our hospital from January 2022 to January 2024. A total of 79 patients were included in the reference group (routine transitional management), and 89 subjects were included in the observation group (smartphone-based hospital-family transitional care). The symptom burden and quality of life in both groups before and after management were compared. The main statistical methods used in this study were the chi-squared test and the Mann-Whitney U test.

**Results:**

Before discharge, no significant difference existed in Geriatric Depression Scale (GDS) scores, P300 latency, P300 amplitude, Montreal Cognitive Assessment (MoCA) scores, and the scores of each domain in the World Health Organization Quality of Life (WHOQOL)-BREF between the two groups (all *p* > 0.05). After 5 months, the observation group demonstrated a significantly lower GDS score (*p* = 0.016), shorter P300 latency (*p* < 0.001), higher P300 amplitude (*p* < 0.001), higher MoCA score (*p* = 0.001), and significantly higher scores in physiological, psychological, and environmental domains than the reference group (*p* < 0.001), with no significant difference in social relation domain (*p* > 0.05).

**Conclusions:**

Smartphone-based hospital-family transitional care can improve the symptom burden, cognitive function, and quality of life of elderly patients with depression.

## Main points

1. Smartphone-based hospital-family transitional care can improve symptom burden in elderly patients with depression.

2. Smartphone-based hospital-family transitional care can improve cognitive function in elderly patients with depression.

3. Smartphone-based hospital-family transitional care can improve quality of life in elderly patients with depression.

## 1. Introduction

Depression is a recurrent neuropsychiatric disease, accompanied by other behavioral defects such as memory impairment [1]. The prevalence of depression is rising, causing a serious global disease burden [2]. The elderly are a high-risk group for depression, who often have coexisting physical diseases, cognitive dysfunction, or both [3]. For example, depression is a risk factor for Alzheimer’s disease [4]. At present, geriatric depression mainly depends on long-term drug control and daily nursing. In fact, elderly patients with depression do not receive adequate attention in routine transitional management. Therefore, it is of great importance to explore better transitional care approaches to improve the disease status of elderly patients with depression.

Transitional care is considered an important therapeutic aspect of mental illness, as it usually involves long-term or recurrent episodes with psychosocial treatment aspects [5]. However, a meta-analysis pointed out that it is currently impossible to determine any convincing effective intervention transition method for patients with depression after psychiatric hospitalization [6]. The evidence for discharge management of depression is limited, heterogeneous, and potentially prone to bias. Although transitional care can detect symptom changes in a timely manner and carry out early management through regular follow-up and evaluation, it may be difficult to achieve sustained and systematic operation in practice due to the need for long-term and systematic intervention. Smartphone-based hospital-family transitional care includes comprehensive evaluation, plan formulation, health monitoring and reminding, and remote monitoring-emergency response. Compared with conventional transitional care, it has the advantages of having diverse functions and providing rich information, and thus meeting the personalized needs of users, whilst improving the efficiency of chronic disease monitoring and promoting physician-patient communication. As smartphone technology further evolves with fifth-generation cellular network expansion, it will play a key role in the future of health medicine, patient referrals, consultation, ergonomics, and many other healthcare applications [7]. In view of its significant advantages, this study suggests that smartphone-based hospital-family transitional care may be more conducive to alleviating the symptom burden and improving the quality of life in elderly patients with depression.

## 2. Materials and Methods

### 2.1 General Data

A total of 168 elderly patients with depression admitted to our hospital from January 2022 to January 2024 were selected for this retrospective study. In accordance with different transitional care modes, 79 patients who underwent routine transitional management were included in the reference group, with two excluded (one case with malignant tumors and one case who could not communicate normally), resulting in 77 cases included. Subsequently, 89 subjects who received smartphone-based hospital-family transitional care were included in the observation group, with five excluded (one case with airway disease, one case with severe infection, two cases with liver and kidney dysfunction, and one case with other mental illness), and 84 cases were finally included.

Inclusion criteria. (1) Patients aged 60–79 years; (2) patients who were diagnosed with depression in our hospital; (3) patients without heart, liver, and kidney failure; (4) patients with a normal intelligence level; and (5) patients without aggressive or suicidal tendencies.

Exclusion criteria. (1) Patients with severe visual or auditory disorders, or could not communicate normally; (2) patients with airway diseases, malignant tumors, or severe infection; and (3) patients with other mental illness.

### 2.2 Methods

#### 2.2.1 Reference Group

The reference group received routine transitional care measures for 5 months. After admission, the medical staff evaluated the patients’ condition, carried out standardized drug intervention, and then perform mental rehabilitation training. During the training process, the family members accompanied patients and mastered the necessary training methods. When the family members had a certain degree of proficiency, nursing staff assisted the family members to carry out independent training for the patients and recorded the daily (30 minutes a day) and weekly (5 days a week) training times. Cognitive training was recommended three times a week, for not less than 30 minutes each time, with a total duration of at least 20 hours of continuous training. After that, nursing staff evaluated the effect of independent training.

After discharge, medical staff regularly implemented out-of-hospital telephone guidance for patients. During this interval, the follow-up staff investigated the family self-care situation of patients, identified time problems, and provided feedback to the nursing staff. Patients were reviewed every month.

#### 2.2.2 Observation Group

Based on the reference group, the observation group was given smartphone-based hospital-family transitional care measures for 5 months. After admission, the hospital immediately set up a WeChat group, invited patients and their families to follow the official accounts, and established the health records for patients.

(1) Through smartphone applications, the medical staff communicated with patients at least five times a week for no less than 30 minutes through video calls, to provide them with emotional support and encouragement, remind them to take medicine on time, and regularly introduce illustrated articles about case analysis, self-care skills, and daily diet to promote reading and facilitate understanding. Meanwhile, existing remote monitoring technology was used to provide telecommunication-based depression nursing management for elderly patients with depression, including symptom monitoring, assessing of antidepressant medication compliance, and dissemination of possible side effects.

(2) The patients’ physiological state and daily living information regarding diet, sleep, and outdoor activities were recorded using the smartphones equipped with health monitoring sensors (for heart rate, blood pressure, and oxygen saturation). The daily data was summarized and fed back to the medical staff before 12:00 pm, and then the medical staff adjusted the service contents and methods according to the feedback to better meet the needs of patients.

(3) Based on the information management platform of family medical care, a home-based transitional care model was constructed. The platform included a monitoring platform and an operation platform, which can realize the comprehensive management of patients.

### 2.3 Observation Indicators

The symptom burden, cognitive function, and quality of life in both groups before discharge and after 5 months of transitional care were compared.

#### 2.3.1 Baseline Data

The baseline data such as sex, age, course of disease, years of education, depression degree, and medical history were collected.

#### 2.3.2 Symptom Burden

The Geriatric Depression Scale (GDS, **Supplementary Table 1**) [8] consisted of 30 items, with 10 items scored in reverse order (a “no” answer indicated the presence of depression) and 20 items scored in positive order (a “yes” answer indicated the presence of depression), with an overall score of 0–30 points (0–10 points: no depression, 11–21 points: mild depression, 21–30 points: severe depression). The reliability of the scale was 0.812, and the structural validity showed single-dimension characteristics.

#### 2.3.3 Cognitive Function

The NDI-094 Haishen electromyography evoked potential instrument (Shanghai Jumu Medical Device Co., Ltd.; Shanghai Medical Products Administration Certified No.: 20192070470; batch No.: JM2029-004555, Shanghai, China) was used for detecting the observation indicators such as P300 latency (healthy population: 312.1 ± 25.5 ms) and P300 amplitude (healthy population: 13.8 ± 5.5 µV), with reference to the international 10–20 electroencephalogram recording system. The recording electrode was placed at the occipital point of patients, the forehead electrode was grounded, and the reference electrode was placed on their two earlobes. Patients relaxed their muscles and stayed awake with eyes closed, waiting for the stimulus. The deviant stimulus was a pure tone at 2000 Hz and 85 dB, with an appearance probability of 0.2 and an interval of 1 s. The subjects were asked to memorize the number of deviant stimuli.

The Montreal Cognitive Assessment (MoCA, **Supplementary Fig. 1**) [9] includes eight items: visual space/executive ability, naming ability, memory ability, attention ability, language fluency, abstract thinking ability, delayed memory ability, and orientation ability. The total score ranges from 0 to 30 points, and <26 was defined as indicative of cognitive impairment. One point was added for ≤12 years of education, with higher scores indicating better cognitive function.

#### 2.3.4 Quality of Life

The World Health Organization Quality of Life-BREF (WHOQOL-BREF,** Supplementary Table 2**) [10] consists of four domains, including physiological, psychological, social relation, and environmental domains, with a total of 26 items. Single items used a 5-level scoring method, and single dimensions were standardized to a percentage system. The total score was the average score of each dimension, with higher scores indicating better quality of life.

### 2.4 Statistical Methods 

Data were processed using SPSS 25.0 (IBM Corp., Armonk, NY, USA). The Shapiro-Wilk method was utilized to test the normal distribution of continuous variables. Data conforming to a normal distribution were expressed as (mean ± standard deviation) and subjected to a *t*-test, while the data not adhering to a normal distribution were represented by (M (P_25_, P_75_)) and tested by the Mann-Whitney U test. *p* < 0.05 was considered statistically significant.

## 3. Results

### 3.1 Baseline Data

No significant differences existed in baseline data between both groups (*p* > 0.05), as shown in Table 1.

**Table 1.** **Comparison of baseline data in both groups**.

Items		Observation group (n = 84)	Reference group (n = 77)	z/*χ*^2^	*p*
Sex	Male	49 (58.33)	46 (59.74)	0.033	0.856
	Female	35 (41.67)	31 (40.26)		
Age (years, M (P_25_, P_75_))		71.00 (67.00, 75.50)	70.00 (66.00, 76.00)	–0.666	0.505
Course of disease (months, M (P_25_, P_75_))		14.00 (12.00, 16.00)	13.00 (11.00, 16.00)	–1.762	0.078
Years of education (years, M (P_25_, P_75_))		7.00 (4.50, 9.00)	8.00 (6.00, 10.00)	–1.680	0.093
Underlying diseases	Coronary heart disease	37 (44.05)	33 (42.86)	0.023	0.879
	Chronic bronchitis	40 (47.62)	36 (46.75)	0.012	0.912
	Hypertension	51 (60.71)	50 (64.94)	0.306	0.580
	Hyperlipidemia	62 (73.81)	55 (71.43)	0.115	0.735
	Diabetes	48 (57.14)	41 (53.25)	0.247	0.619
Depression degree	Mild	20 (23.81)	18 (23.38)	0.038	0.981
	Moderate	39 (46.43)	35 (45.45)		
	Severe	25 (29.76)	24 (31.17)		
Accompanying person	Spouses	29 (34.52)	30 (38.96)	0.775	0.679
	Children	34 (40.48)	32 (41.56)		
	Nursing workers	21 (25.00)	15 (19.48)		
Marital status	Married	55 (65.48)	51 (66.23)	0.010	0.919
	Divorced/widowed	29 (34.52)	26 (33.77)		
Payment methods	Medical insurance	68 (80.95)	60 (77.92)	0.226	0.634
	Self-pay	16 (19.05)	17 (22.08)		

### 3.2 Symptom Burden 

Before discharge, there was no significant difference in GDS scores between the two groups (*p* > 0.05). After 5 months, the observation group showed significantly lower GDS score than the reference group (*p* < 0.05), see Table 2.

**Table 2.** **Comparison of GDS scores in both groups (points, M (P_25_, P_75_))**.

Groups	Observation group (n = 84)	Reference group (n = 77)	z	*p*
Before discharge	22.00 (20.00, 25.00)	23.00 (19.00, 26.00)	–0.909	0.363
After 5 months	14.50 (10.00, 18.50)	16.00 (13.00, 20.00)	–2.408	0.016

GDS, Geriatric Depression Scale.

### 3.3 Cognitive Function

Before discharge, no significant difference existed in P300 latency, P300 amplitude, and MoCA scores between the two groups (*p* > 0.05). After 5 months, the observation group demonstrated significantly shorter P300 latency, higher P300 amplitude, and higher MoCA score (*p* < 0.05), as detailed in Table 3.

**Table 3.** **Comparison of P300 latency, P300 amplitude, and MoCA scores in both groups (M (P_25_, P_75_))**.

Groups		Observation group (n = 84)	Reference group (n = 77)	z	*p*
P300 latency (ms)	Before discharge	350.65 (346.30, 356.05)	351.40 (347.20, 356.20)	–0.459	0.647
	After 5 months	327.00 (320.70, 330.95)	336.50 (332.30, 341.20)	–8.588	<0.001
P300 amplitude (µv)	Before discharge	6.55 (5.30, 7.65)	6.60 (5.60, 8.00)	–1.011	0.312
	After 5 months	10.70 (8.85, 12.00)	9.00 (7.80, 10.30)	–5.284	<0.001
MoCA (points)	Before discharge	18.00 (14.00, 23.00)	17.00 (12.00, 23.00)	–1.302	0.193
	After 5 months	23.00 (20.00, 27.00)	21.00 (17.00, 24.00)	–3.283	0.001

MoCA, Montreal Cognitive Assessment.

### 3.4 Quality of Life

Before discharge, both groups had no significant difference in the scores of each domain of the WHOQOL-BREF (*p* > 0.05). After 5 months, the scores of the physiological, psychological, and environmental domains in the observation group were significantly higher than those in the reference group (*p* < 0.001), with no significant difference in the social relation domain (*p* > 0.05); see Table 4.

**Table 4.** **Comparison of WHOQOL-BREF scores in both groups (points, M (P_25_, P_75_))**.

Groups		Observation group (n = 84)	Reference group (n = 77)	z	*p*
Physiological domain	Before discharge	44.00 (41.00, 47.00)	43.00 (39.00, 47.00)	–1.427	0.154
	After 5 months	56.00 (51.00, 60.00)	51.00 (47.00, 56.00)	–4.942	<0.001
Psychological domain	Before discharge	49.50 (46.00, 52.50)	49.00 (44.00, 52.00)	–0.927	0.354
	After 5 months	58.00 (54.00, 62.00)	53.00 (51.00, 58.00)	–4.728	<0.001
Social relation domain	Before discharge	42.00 (40.00, 44.00)	41.00 (39.00, 44.00)	–1.386	0.166
	After 5 months	46.00 (43.00, 50.00)	46.00 (43.00, 49.00)	–0.372	0.710
Environmental domain	Before discharge	49.00 (44.50, 53.00)	48.00 (45.00, 53.00)	–0.485	0.628
	After 5 months	58.50 (54.00, 62.00)	55.00 (52.00, 59.00)	–3.583	<0.001

WHOQOL-BREF, World Health Organization Quality of Life-BREF.

## 4. Discussion

Geriatric depression is a growing global problem due to demographic changes [11]. At present, there is no radical treatment for geriatric depression in clinical practice. Depression often recurs in elderly patients, placing a heavy burden on themselves and their families. Improving the quality of care outside the hospital will therefore undoubtedly bring great benefits for patients. The current addition of smartphones and wearable devices to clinical trials offers a unique opportunity to monitor behavior in a non-invasive manner [12]. This study suggests that smartphone-based hospital-family transitional care can effectively improve the symptom burden, cognitive function, and quality of life of elderly patients with depression, through the effects of efficacious management. These contributions improve the efficiency and quality of care, promote the combination of medical and health care, and provide new ideas and methods for future medical care.

Geriatric depression is related to heavy disease burden [13]. The results of the present study demonstrated that the GDS score in the observation group was significantly lower than that in the reference group after 5 months of transitional care, indicating that smartphone-based hospital-family transitional care can effectively improve the symptom burden of elderly patients and reduce the impact of depression on them. This is because smartphone-based transitional care improves the quality of care and promotes the effective use of nursing resources. Smartphones make medical consultation and rehabilitation guidance more concrete, further effectively meeting the needs of elderly patients and reducing their symptom burden. The study of Kim* et al*. [14] has shown that awareness of stroke, depression, and blood pressure is enhanced when using smartphone-based mobile healthcare systems, which is similar to the results of this study.

Cognitive impairment is one of the common accompanying symptoms of depression [15]. Event-related potentials are common in most electrophysiological recordings in which the P300 wave is regarded as the best indicator of mental state [16]. Changes in P300 latency and amplitude, and neuropsychological testing are conducive to detecting the early signs of mild impairments in higher cognitive processing [17]. The present study revealed that no significant difference existed in P300 latency and P300 amplitude between the two groups before discharge, while the observation group demonstrated significantly shorter P300 latency and higher P300 amplitude after 5 months, indicating that smartphone-based transitional care can improve the mental retardation and cognitive decline of elderly patients. The reason may be that based on smartphones, medical staff can effectively carry out psychological counseling for patients and deal with their problems in a timely manner, which has a remarkable effect on enhancing the cognitive function of patients. After 5 months, the observation group had a significantly higher MoCA score than the reference group, which also substantiates this point.

A previous study confirmed that smartphone-based guided aftercare interventions are highly accepted by patients, and may support rehabilitation after hospitalization, thereby improving the aftercare of patients with neurological bulimia [18]. The present study found that after 5 months, the scores of physiological, psychological, and environmental domains in the observation group were significantly higher than those in the reference group (*p* < 0.001), with no significant difference in the social relation domain (*p* > 0.05), indicating that smartphone-based transitional care can improve the quality of life of elderly patients with depression, but that it has no significant effect on the social relation domain. It has been shown that smartphones can facilitate doctor-patient collaboration through WeChat and other health platforms. In addition to systematic health education guidance, elderly patients with depression are guided to carry out rehabilitation training and take medicine on time, ultimately improving the treatment effect and quality of life. Pai* et al*. [19] have also pointed out that medical staff should provide patients with individual transitional care, encourage them to participate in community health promotion activities, and strengthen the function of daily living ability to improve the quality of life.

Our study has some limitations. Firstly, the sample size was small, and may not be fully representative of the entire population, thereby potentially reducing the generalizability of the conclusions. Secondly, this study involves out-of-hospital management and therefore the obtained data may be biased. Finally, the follow-up time of this study was only 5 months, and no long-term dynamic tracking was carried out. Therefore, in the future, a prospective study with a larger sample should be carried out to provide reference for transitional care in elderly patients with depression. At the same time, future research should also strengthen interdisciplinary cooperation and combine medicine, psychology, information technology, and other fields to promote the development of smartphone-based hospital-family transitional care in elderly patients with depression.

## 5. Conclusions

Smartphone-based hospital-family transitional care can effectively improve the symptom burden, cognitive function, and quality of life of elderly patients, and help them to reintegrate into normal family life.

## Availability of Data and Materials

The original contributions presented in the study are included in the article. Further inquiries can be directed to the corresponding authors.

## Supplementary Material

Supplementary material associated with this article can be found, in the online version, at https://doi.org/10.31083/AP39894.
