# Supplementary material for: Effects of Smartphone-Based Hospital-Family Transitional Care on Symptom Burden and Quality of Life in Elderly Patients with Depression
Source: Alpha Psychiatry. 2025 Apr 22;26(2):39894. doi: 10.31083/AP39894 (PMC12059787; doi:10.31083/AP39894)
Supplement: Supplementary file 1 [file 2757-8038-26-2-39894-s1.docx]

Supplementary Table 1. Geriatric Depression Scale (GDS).

|  | Questions | Answers | | Points |
| --- | --- | --- | --- | --- |
| 1 | Are you basically satisfied with your life? | Yes | No |  |
| 2 | Have you dropped many of your activities and interests? | Yes | No |  |
| 3 | Do you feel that your life is empty? | Yes | No |  |
| 4 | Do you often get bored? | Yes | No |  |
| 5 | Do you see any hope for the future? | Yes | No |  |
| 6 | Are you annoyed that you can't get rid of some thoughts in your head? | Yes | No |  |
| 7 | Are you in good spirits most of the time? | Yes | No |  |
| 8 | Are you afraid that something bad is going to happen to you? | Yes | No |  |
| 9 | Do you feel happy most of the time? | Yes | No |  |
| 10 | Do you often feel helpless? | Yes | No |  |
| 11 | Do you often feel upset? | Yes | No |  |
| 12 | Do you prefer to stay at home, rather than going out and doing new things? | Yes | No |  |
| 13 | Do you often worry about the future? | Yes | No |  |
| 14 | Do you feel you have more problems with memory than most? | Yes | No |  |
| 15 | Do you think it is wonderful to be alive? | Yes | No |  |
| 16 | Do you often feel depressed? | Yes | No |  |
| 17 | Do you feel pretty worthless the way you are now? | Yes | No |  |
| 18 | Do you always worry about the past? | Yes | No |  |
| 19 | Do you find life exciting? | Yes | No |  |
| 20 | Is it difficult for you to start a new job? | Yes | No |  |
| 21 | Do you feel full of energy? | Yes | No |  |
| 22 | Do you feel that your situation is hopeless? | Yes | No |  |
| 23 | Do you think that most people are better off than you are? | Yes | No |  |
| 24 | Do you often feel sad about small things? | Yes | No |  |
| 25 | Do you often feel like crying? | Yes | No |  |
| 26 | Do you have trouble concentrating? | Yes | No |  |
| 27 | Do you feel happy when you wake up in the morning? | Yes | No |  |
| 28 | Do you want to avoid gatherings? | Yes | No |  |
| 29 | Is it easy for you to make decisions? | Yes | No |  |
| 30 | Is your mind as clear as usual? | Yes | No |  |

Supplementary Fig 1. Montreal Cognitive Assessment (MoCA).


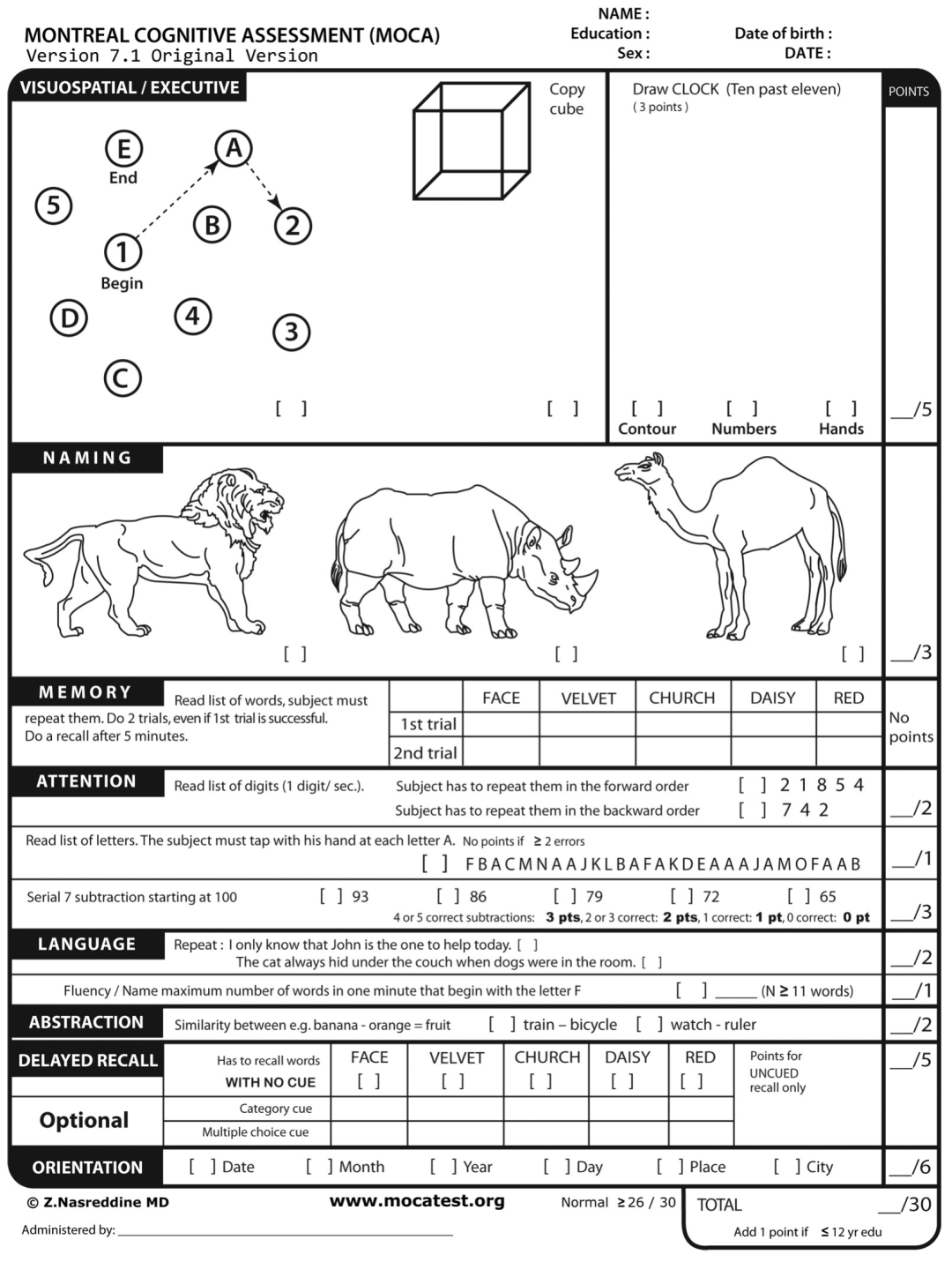


Supplementary Table 2. The World Health Organization Quality of Life-BREF (WHOQOL-BREF).

|  | Questions | Scoring standards |
| --- | --- | --- |
| 1 | How do you evaluate your quality of life? | 1 point-very poor; 2 points-poor; 3 points-not good or bad; 4 points-good; 5 points-very good |
| 2 | Do you satisfied with your health? | 1 point-very dissatisfied; 2 points-dissatisfied; 3 points-neither satisfied nor dissatisfied; 4 points-satisfied; 5 points-very satisfied |
| 3 | Do you think the pain is preventing you from doing what you need to do? | 1 point-not at all; 2 points-slightly; 3 points-nearly; 4 points-quite; 5 points-extremely |
| 4 | Do you need medical help in daily life? | 1 point-not at all; 2 points-slightly; 3 points-nearly; 4 points-quite; 5 points-extremely |
| 5 | Do you find life enjoyable? | 1 point-not at all; 2 points-slightly; 3 points-nearly; 4 points-quite; 5 points-extremely |
| 6 | Do you think your life is meaningful? | 1 point-not at all; 2 points-slightly; 3 points-nearly; 4 points-quite; 5 points-extremely |
| 7 | Can you concentrate? | 1 point-not at all; 2 points-slightly; 3 points-nearly; 4 points-quite; 5 points-extremely |
| 8 | Do you feel secure in your daily life? | 1 point-not at all; 2 points-slightly; 3 points-nearly; 4 points-quite; 5 points-extremely |
| 9 | Is your living environment good for health? | 1 point-very poor; 2 points-poor; 3 points-not good or bad; 4 points-good; 5 points-very good |
| 10 | Do you have sufficient energy in daily life? | 1 point-not at all; 2 points-slightly; 3 points-nearly; 4 points-quite; 5 points-extremely |
| 11 | Do you think your appearance is acceptable? | 1 point-not at all; 2 points-slightly; 3 points-nearly; 4 points-quite; 5 points-extremely |
| 12 | Do you have enough money? | 1 point-not at all; 2 points-slightly; 3 points-nearly; 4 points-quite; 5 points-extremely |
| 13 | Do you have all the information you need in your daily life? | 1 point-not at all; 2 points-slightly; 3 points-nearly; 4 points-quite; 5 points-extremely |
| 14 | Do you have the opportunity to engage in leisure activities? | 1 point-not at all; 2 points-slightly; 3 points-nearly; 4 points-quite; 5 points-extremely |
| 15 | How is your mobility? | 1 point-very poor; 2 points-poor; 3 points-not good or bad; 4 points-good; 5 points-very good |
| 16 | Are you satisfied with your sleep situation? | 1 point-very dissatisfied; 2 points-dissatisfied; 3 points-neither satisfied nor dissatisfied; 4 points-satisfied; 5 points-very satisfied |
| 17 | Are you satisfied with your ability to do daily tasks? | 1 point-very dissatisfied; 2 points-dissatisfied; 3 points-neither satisfied nor dissatisfied; 4 points-satisfied; 5 points-very satisfied |
| 18 | Are you satisfied with your work ability? | 1 point-very dissatisfied; 2 points-dissatisfied; 3 points-neither satisfied nor dissatisfied; 4 points-satisfied; 5 points-very satisfied |
| 19 | Are you satisfied with yourself? | 1 point-very dissatisfied; 2 points-dissatisfied; 3 points-neither satisfied nor dissatisfied; 4 points-satisfied; 5 points-very satisfied |
| 20 | Are you satisfied with your interpersonal relationships | 1 point-very dissatisfied; 2 points-dissatisfied; 3 points-neither satisfied nor dissatisfied; 4 points-satisfied; 5 points-very satisfied |
| 21 | Are you satisfied with your sexual life? | 1 point-very dissatisfied; 2 points-dissatisfied; 3 points-neither satisfied nor dissatisfied; 4 points-satisfied; 5 points-very satisfied |
| 22 | Are you satisfied with the support you received from your friends? | 1 point-very dissatisfied; 2 points-dissatisfied; 3 points-neither satisfied nor dissatisfied; 4 points-satisfied; 5 points-very satisfied |
| 23 | Are you satisfied with the conditions of your residence? | 1 point-very dissatisfied; 2 points-dissatisfied; 3 points-neither satisfied nor dissatisfied; 4 points-satisfied; 5 points-very satisfied |
| 24 | Are you satisfied with the convenience of accessing healthcare services? | 1 point-very dissatisfied; 2 points-dissatisfied; 3 points-neither satisfied nor dissatisfied; 4 points-satisfied; 5 points-very satisfied |
| 25 | Are you satisfied with your transportation situation? | 1 point-very dissatisfied; 2 points-dissatisfied; 3 points-neither satisfied nor dissatisfied; 4 points-satisfied; 5 points-very satisfied |
| 26 | Do you have negative feelings (such as despair, anxiety, depression) | 1 point-not at all; 2 points-slightly; 3 points-nearly; 4 points-quite; 5 points-extremely |
| 27 | Does domestic dissension affect your life? | 1 point-not at all; 2 points-slightly; 3 points-nearly; 4 points-quite; 5 points-extremely |
| 28 | How is your appetite? | 1 point-very poor; 2 points-poor; 3 points-not good or bad; 4 points-good; 5 points-very good |
| 29 | If you were asked to give a total score for your quality of life based on the above aspects (physical health, mental health, social relationships, and surrounding environment, etc.), how would you rate it? (Out of 100 points) | |
